# Efficient integrated production of bioethanol and antiviral glycerolysis lignin from sugarcane trash

**DOI:** 10.1186/s13068-023-02333-z

**Published:** 2023-05-15

**Authors:** Sadat Mohamed Rezk Khattab, Hiroyuki Okano, Chihiro Kimura, Takashi Fujita, Takashi Watanabe

**Affiliations:** 1 Research Institute for Sustainable Humanosphere, Kyoto University, Gokasho, Uji, Kyoto 611-0011 Japan; 2 Faculty of Science, Al-Azhar University, Assiut, 71524 Egypt; 3 Institute for Frontier Life and Medical Sciences, Kyoto University, Shogoin, Kawahara-Cho, Sakyo-Ku, Kyoto, 606-8507 Japan

**Keywords:** Integrated biorefinery, Sugarcane trash, Microwave acidic glycerolysis, Antiviral, Glycerol conversion, Bioethanol, *Saccharomyces cerevisiae*

## Abstract

**Background:**

Sugarcane trash (SCT) represents up to 18% of the aboveground biomass of sugarcane, surpassing 28 million tons globally per year. The majority of SCT is burning in the fields. Hence, efficient use of SCT is necessary to reduce carbon dioxide emissions and global warming and establish agro-industrial biorefineries. Apart from its low costs, conversion of whole biomass with high production efficiency and titer yield is mandatory for effective biorefinery systems. Therefore, in this study, we developed a simple, integrated method involving a single step of glycerolysis pretreatment to produce antiviral glycerolysis lignin (AGL). Subsequently, we co-fermented glycerol with hydrolyzed glucose and xylose to yield high titers of bioethanol.

**Results:**

SCT was subjected to pretreatment with microwave acidic glycerolysis with 50% aqueous (aq.) glycerol (MAG_50_); this pretreatment was optimized across different temperature ranges, acid concentrations, and reaction times. The optimized MAG_50_ (^op^MAG_50_) of SCT at 1:15 (w/v) in 1% H_2_SO_4,_ 360 µM AlK(SO_4_)_2_ at 140 °C for 30 min (^op^MAG_50_) recovered the highest amount of total sugars and the lowest amount of furfural byproducts. Following ^op^MAG_50_, the soluble fraction, i.e., glycerol xylose-rich solution (GXRS), was separated by filtration. A residual pulp was then washed with acetone, recovering 7.9% of the dry weight (27% of lignin) as an AGL. AGL strongly inhibited the replication of encephalomyocarditis virus (EMCV) in L929 cells without cytotoxicity. The pulp was then saccharified in yeast peptone medium by cellulase to produce a glucose concentration similar to the theoretical yield. The total xylose and arabinose recoveries were 69% and 93%, respectively. GXRS and saccharified sugars were combined and co-fermented through mixed cultures of two metabolically engineered *Saccharomyces cerevisiae* strains: glycerol-fermenting yeast (SK-FGG4) and xylose-fermenting yeast (SK-N2). By co-fermenting glycerol and xylose with glucose, the ethanol titer yield increased to 78.7 g/L (10% v/v ethanol), with a 96% conversion efficiency.

**Conclusion:**

The integration of AGL production with the co-fermentation of glycerol, hydrolyzed glucose, and xylose to produce a high titer of bioethanol paves an avenue for the use of surplus glycerol from the biodiesel industry for the efficient utilization of SCT and other lignocellulosic biomasses.

**Supplementary Information:**

The online version contains supplementary material available at 10.1186/s13068-023-02333-z.

## Background

The development of effective biorefinery technologies based on renewable plant biomass is inevitable for meeting the high energy demand from biofuels and bioproducts. The use of a lignocellulosic biorefinery helps mitigate the climate change crisis while also revitalizing the rural economy [1–4]. Annually, more than 100 billion tons of dry lignocellulosic biomass are photosynthesized [5]. Sugarcane (*Saccharum officinarum*) is an essential crop used worldwide, particularly in tropical areas. Annual sugarcane production exceeds 1.6 billion tons, resulting in hundreds of million metric tons of biomass leftovers [6–8]. Sugarcane trash (SCT), which refers to the upper shoots and the leaf sheath with the leaves that are left on the ground, accounts for approximately 15% of the total aboveground biomass. Approximately 10–17 tons of dry matter per hectare is estimated. Owing to the low profit margins of traditional SCT use, field burning has been the primary method for SCT disposal, resulting in biological and environmental problems [9–11]. SCT is a voracious carbon capture and storage model that does not compete with food chains or land cultivation. Therefore, the exploitation of SCT has attracted considerable attention in the production of bioproducts, such as bioethanol and other value-added goods.

To produce biofuels and chemicals, SCT has been pretreated using various methods, such as microbial degradation [12], milling [13], acidolysis [14–16], alkali-based ammonia fiber expansion [10], surfactant-assisted ultrasound pretreatment [17], alkali degradation [14, 18], and organosolvolysis with acids or transition metals [7, 19, 20]. Conventional thermochemical biorefinery processes are expensive [21]. Conversely, compared with traditional heating, microwave heating can decrease the conversion cost and energy input [11, 22, 23]. The heating efficiency increases when solvents with high permittivity, such as glycerol and ethylene glycol, are used. For socially implementing microwave pretreatment of biomass, we developed a highly efficient pilot-plant-scale microwave irradiation reactor (50 L) with a simple low reflection for woody biomass pretreatment [24].

The surplus glycerol produced by the biodiesel industry is a major impediment to its expansion and sustainability [25]. It is a renewable byproduct of the plant oil industry [3]. As a solvent, glycerol has several advantages. It is affordable, nontoxic, has high permittivity, and is less inhibitory to enzymatic hydrolysis and fermentation [3]. Glycerol has received considerable attention as a cost-effective material for the biorefinery of lignocelluloses, such as sugarcane, softwood, and hardwood, during pretreatment with and without acid catalysts including AlK(SO_4_)_2_ (alum) and other Lewis acids using conventional and microwave heating [3, 26–29]. After pretreatment, glycerol was used as a carbon source to produce microbial oils in previous studies [28, 29]. Recently, we developed an efficient glycerol-converting yeast strain by genetically modifying the oxidation of cytosolic NADH through an O_2_-dependent dynamic shuttle and abolishing both glycerol phosphorylation and biosynthesis in *Saccharomyces cerevisiae* as well as by the vigorous expression of whole genes in the dihydroxyacetone pathway [30]. Furthermore, we previously reported the enhancement of fermentation of xylose to bioethanol using recombinant *S. cerevisiae*, which improved the recycling of NADP^+^/NADPH via a xylose oxidoreductase pathway [31, 32].

The low titer and yield of bioethanol produced from biomass are key factors restricting its use on an industrial scale. High solid loading of residual cellulose-rich solids after pretreatment is a frequently reported remedy to overcome titer productivity [33, 34]. To recover hemicellulose sugars with this approach, additional steps are required, such as the use of a countercurrent belt filter to reduce sugar dilution and eliminate soluble inhibitors generated during pretreatment as well as a solvent recovery step [33, 34]. Therefore, in the present study, we developed a method for producing a high titer yield of ethanol and antiviral glycerolysis lignin (AGL) to avoid the complexity of sugar separation and solvent recovery and their associated costs while maintaining the high titer productivity of bioethanol. This integrated biorefinery method contributes toward the industrial application of SCT, linking the biodiesel, bioethanol, and pharmaceutical industries.

## Results and discussion

**Fig. 1:**
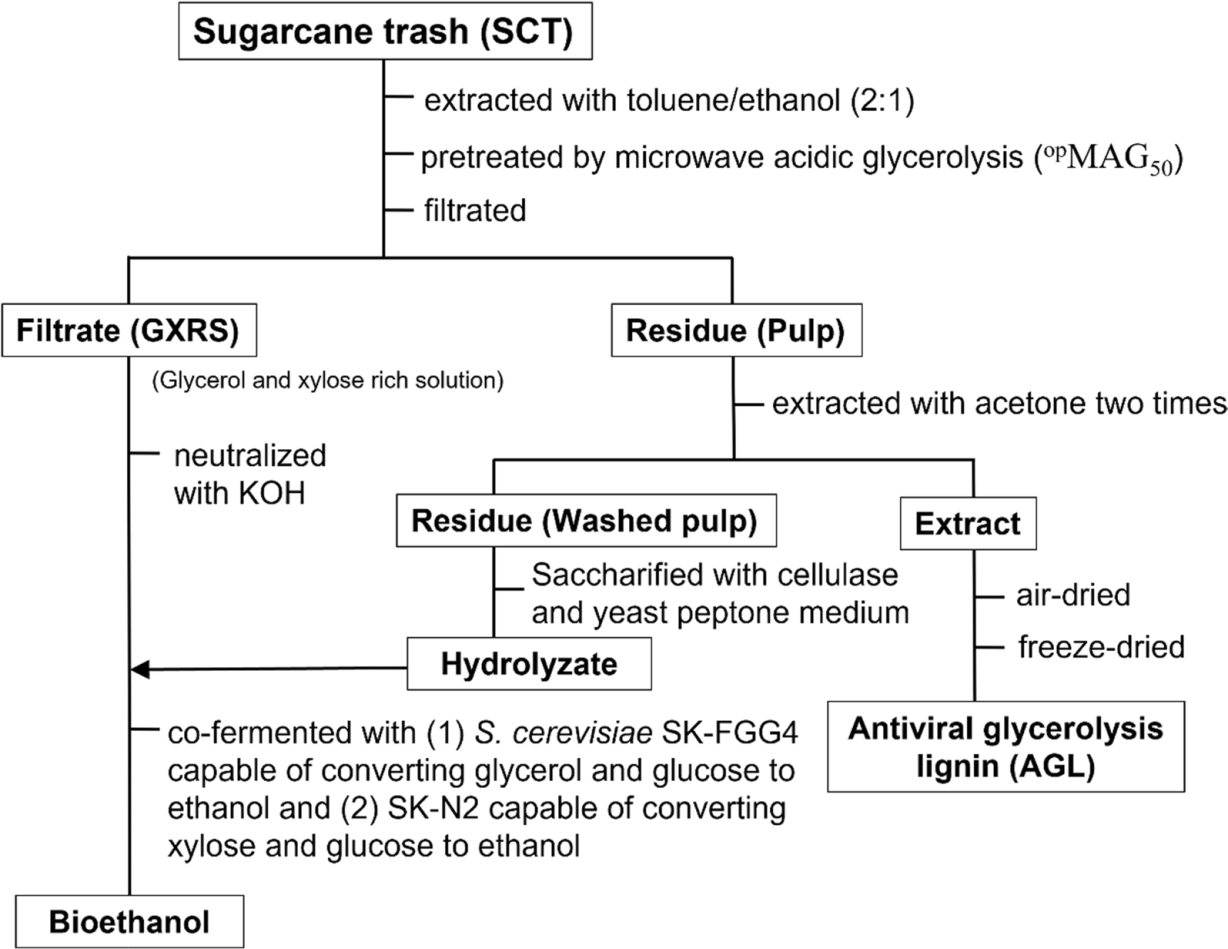
Schematic of the integrated sugarcane trash biorefinery that produces bioethanol and AGL using ^op^MAG_50_ pretreatment and recombinant yeasts

Multiple bioprocesses, low titer yield, and high overall cost represent a barrier to bioethanol biorefinery from SCT. To develop an effective biorefinery, we used glycerol as a solvent in ^op^MAG_50_ to enhance the delignification of lignin, generate AGL, and boost the saccharification efficiency (Fig. 1). Subsequently, glycerol was co-fermented with GXRS filtrate sugars and the hydrolyzed sugars of saccharification to produce a high bioethanol yield of bioethanol by using mixed precultures of (1) *S. cerevisiae* SK-FGG4 capable of converting glycerol and glucose to ethanol [30] and (2) SK-N2 capable of converting xylose and glucose [31], as illustrated in Fig. 1. In this scenario, simply rinsing the residual pulp of ^op^MAG_50_ with acetone, not only saccharification produced a similar theoretical yield, but also generated AGL against a nonenveloped virus, the encephalomyocarditis virus (EMCV), without detectable cytotoxicity. Furthermore, fermentation of the mixture of GXRS filtrate and hydrolyzed pulp sugars without any purification produced a high bioethanol yield, theoretically equal to the cost-effectiveness of the industrial distillation process (Fig. 1).

### Compositional analysis of sugarcane trash

The compositional analysis of native SCT (mg/g) is shown in Table 1 as follows: glucose (335 ± 07), xylose (163 ± 10), arabinose (28 ± 04), lignin (293 ± 17), ash (129 ± 17), extractives (54.3 ± 2), and moisture content (92.5 ± 1.2). The determination procedures are detailed in the Methods section. These compositional contents are within the range of previously published reports [12–21], despite the slightly higher ash content in this study.

**Table 1:** Sugar recoveries from extractives-free SCT by ^op^MAG_50_ and pulp enzymatic saccharification, compared with those of the theoretical yield. Data are presented based on the dry weight

Compositional content of SCT (mg/g)	Sugar recoveries (mg/g)				
	Theoretical yield	^op^MAG_50_	ES^a^	Total	Efficiency (%)
Glucose	335 ± 07	032 ± 04	298 ± 08	330 ± 12	099 ± 00.2
Xylose	163 ± 10	093 ± 08	020 ± 01	113 ± 09	069 ± 0.04^b^
Arabinose	028 ± 04	026 ± 04	ND^c^	026 ± 04	093 ± 00.1^b^
Total sugars	525 ± 21	151 ± 16	318 ± 09	469 ± 25	089 ± 00.4

Lignin, ash, mineral extract, and moisture contents of native SCT were 293 ± 17, 129 ± 17, 54.3 ± 2, and 92.5 ± 1.2 (mg/g), respectively

^a^Enzymatic saccharification was conducted using 8-cell filter paper units (FPU) of Cellic^®^ CTec2 in 50 mM succinic buffer (pH 5) supplemented with yeast extract (10 g/L) and peptone (20 g/L) in the presence of 250 µg/L aureobasidin A

^b^Conversion efficiencies of C5 increased to 84% when the furfural ratio was 22.3 mg/g. Subsequently, the total recovery of holocellulose reached 94%

^c^Not detected

### Optimization scheme of MAG_50_ for SCT

In this experiment, extractives-free SCT was used to avoid the interference of extractives. Consistent with our previous report [27] and to recover the highest amount of total sugars from GXRS and saccharification of the residual pulp while generating the lowest degradation of xylose to furfural (FF) in the current study, the experiments were performed across a range of temperatures (100–150 °C), times (25–50 min), and acid contents of 360 µM alum with 1–0.5% H_2_SO_4_.

### Effect of MAG_50_ temperatures on SCT

**Fig. 2:**
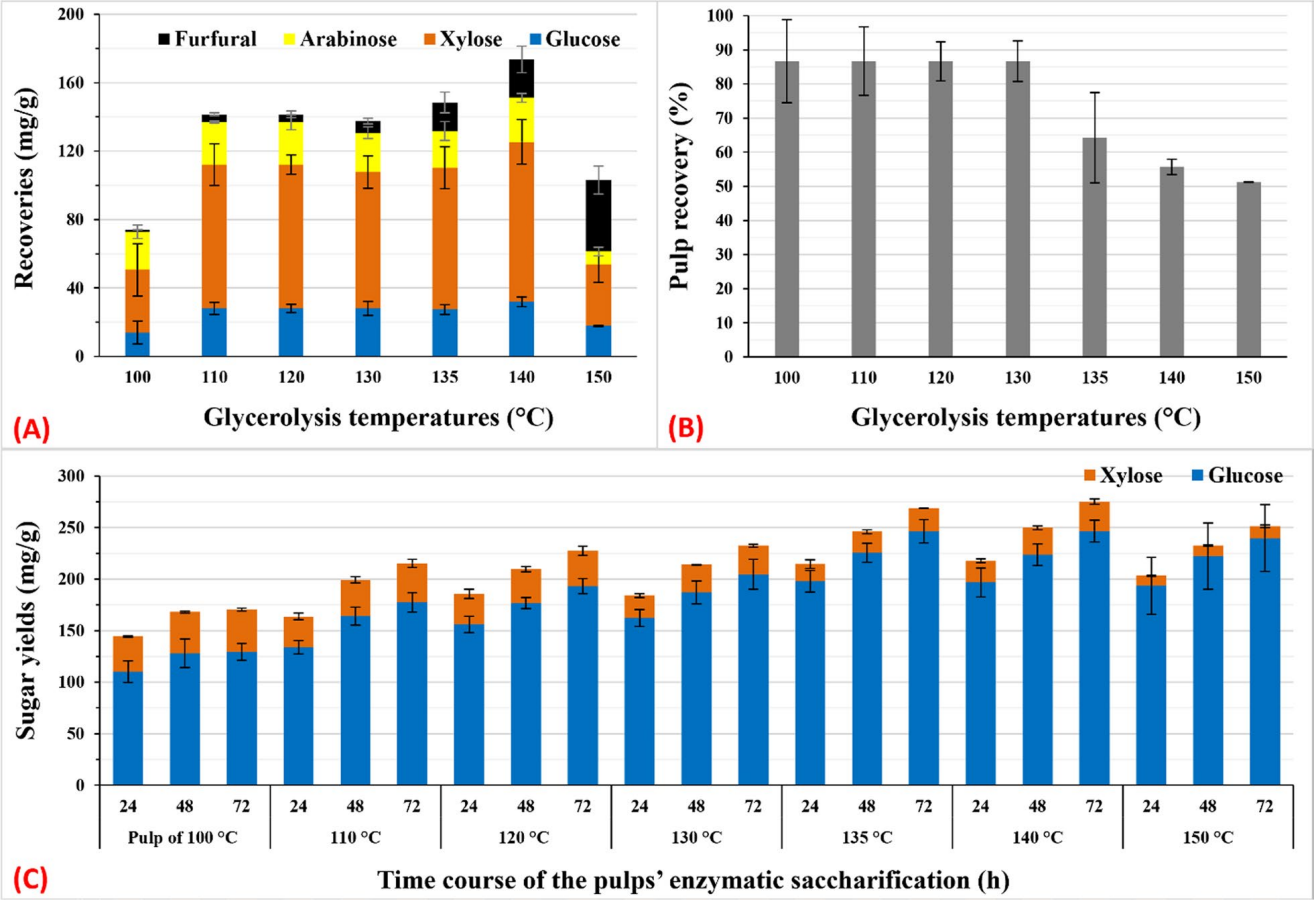
Effects of MAG_5_ temperatures on sugar recovery, pulp yield of SCT, and saccharification of the pulp. Sugar recovery and furfural concentration in GXRS (**A**), pulp yield (**B**), and sugar yield from saccharification of pulp by 10 FPU cellulase (**C**). The pretreatment reaction 1/15 (w/v) was performed within the range of 100–150 °C for 30 min with 50% aq. glycerol, 360 µM alum, and 1.0% H_2_SO_4_. Data represent the average value of three experiments. Error bars are the standard deviation (SD) from the mean value

The temperature during MAG_50_ has a substantial effect on total sugar recovery and residual pulp (Fig. 2a, b). At 1/15 (w/v) of biomass loading, MAG_50_ at 100 °C solubilized 14% of the total sugars of the SCT, which increased to 27% at 110 °C. MAG_50_ was performed at 140 °C, which solubilized 33% of the total sugars with the concomitant production of 4.9% FF (Fig. 2a). Subsequently, when the temperature was increased to 150 °C, the released monomeric sugars decomposed, decreasing the total sugar yield to 19.6%, with the concomitant production of 7.8% FF (Fig. 2a). Additionally, FF production increased with an increase in temperature, which is consistent with the findings of previous studies [35, 36]. We did not detect hydroxymethylfurfural (HMF), the degradation product of glucose, although the glucose level decreased (Fig. 2a). Furthermore, the FF production at 150 °C was not consistent even with the xylose released at 140 °C (Fig. 2a). It is well known that FF can be polymerized to humin [35]. Moreover, prolonged temperature and time during acid pretreatment produced FF and its degradation products, such as organic acids (e.g., formic acid) and pseudo-lignin [36, 37]. Therefore, the loss of mass balance at 150 °C (Fig. 1a) can be attributed to the formation of such degradation products, which is outside the scope of this study. Figure 2b presents the SCT hemicellulose sugars solubilized in the range of 110–130 °C during MAG_50_ at 30 min of reaction_._ This range of temperatures also slightly generated FF (Fig. 2a). Conversely, the amount of pulp decreased significantly when the temperature during MAG_50_ was > 130 °C, indicating the partial solubilization of lignin. The pulp was retained stable at 86.7% of the original weight at 100–130 °C and then gradually decreased to 64.2%, 55.7%, and 51.2% at 135 °C, 140 °C, and 150 °C, respectively (Fig. 2b). As a result of enhanced delignification with hemicellulose solubilization, saccharification was significantly enhanced at 135 °C and 140 °C (Fig. 2c). When MAG_50_ was performed at 140 °C for 30 min, the solubilization level of lignin reached 49%, which enhanced saccharification, facilitating the recovery of 81.1% of the theoretical recovery (Fig. 2a, c). We observed a similar result with acid-catalyzed glycerolysis of sugarcane bagasse, despite the different pretreatment conditions [29]. Unless a partial delignification occurs, it is unlikely that the solubilization of SCT hemicellulose will be sufficient for effective saccharification (MAG_50_ at 100–130 °C versus MAG_50_ at 135–140 °C in Fig. 2). These findings are also supported by previously reported data [3, 26–29]. Furthermore, pore formation in Eucalyptus chips was induced by partially removing hemicellulose and lignin via cooking; this was performed before the ball mill recovered the theoretical digestibility of glucan [38], which supports the findings regarding low glucose and xylose recovery from the enzymatic saccharification of ball and wet disk milling of SCT at 77.6% and 56.8% and 68.0% and 44.9%, respectively [13].

### Effects of MAG_50_ reaction times

**Fig. 3:**
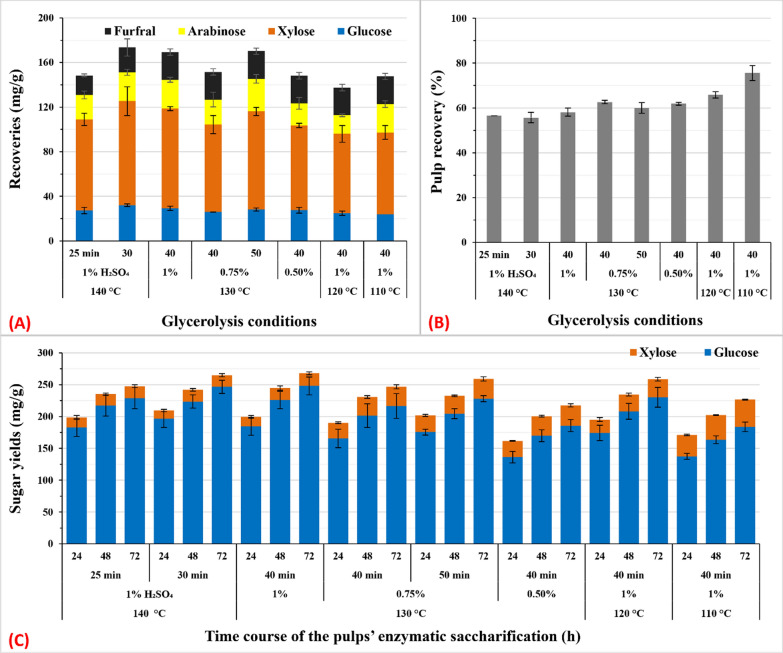
Effects of MAG_50_ reaction time on sugar recovery and furfural concentration in GXRS (**A**), pulp yield (**B**) and sugar yield after enzymatic saccharification of pulp with 10 FPU cellulase (**C**). The pretreatment reaction 1/15 (w/v) was performed within the range of 110–140 °C for 25–50 min with 50% aq. glycerol, 360 µM alum, and 0.5–1.0% H_2_SO_4_. Data represent the average value of three experiments. Error bars are the standard deviation (SD) from the mean value

FF is not only a byproduct in this study, but also an inhibitor substrate for *S. cerevisiae* during either cultivation or fermentation. We found that MAG_50_ at 140 °C resulted in the highest total recovery; however, 14% of xylose was degraded and later detected as FF (Fig. 2). The potential of changing reaction times to reduce the production of FF while maintaining high sugar recoveries was studied and monitored (Fig. 3). Because decreasing the reaction time decreases FF production [35], the MAG_50_ reaction time at 140 °C was reduced to 25 min and compared with 30 min. The results showed that the amount of FF decreased by 22% but that of total sugars decreased by 9% (Fig. 3a, c). The amount of residual pulp increased slightly by 2% (Fig. 3b). Conversely, an increase in the reaction time at 130 °C from 30 to 40 min increased the total sugar recovery and FF production by 10% and 11%, respectively (Figs. 2, 3). We also assessed the effects of a 50-min reaction compared with 40 min while reducing H_2_SO_4_ concentration by 25% to avoid severe degradation (Fig. 3). Because of this extension of time, an improvement of 8% in the total sugar recovery and FF production was observed (Fig. 3a, c); the amount of residual pulp decreased by 2.6% (Fig. 3b). At 110 °C and 120 °C, a 10-min increase in reaction time only enhanced xylose degradation to FF (by 5.5-fold), whereas the total sugar recovery did not change significantly (Figs. 2, 3). It was reported that prolonging the reaction temperature and acid pretreatment time of corncob could degrade hemicellulose monomers to produce FF and organic acids such as formic acid [38].

### Effect of decreasing acidity during MAG_50_ at 140 °C and 150 °C

**Fig. 4:**
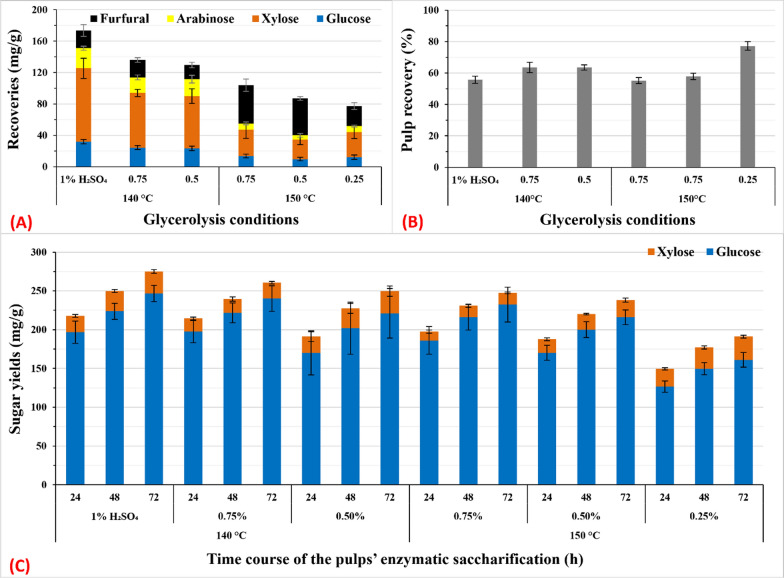
Effects of different concentrations of H_2_SO_4_ during MAG_50_ pretreatment on sugar recovery and furfural concentrations in GXRS (**A**), pulp yield (**B**), and sugar yield after enzymatic saccharification of pulp with 10 FPU cellulase (**C**). The pretreatment reaction of SCT 1/15 (w/v) was performed at 140 °C and 150 °C for 30 min with 50% aq. glycerol, 360 µM alum, and 0.25–1.0% H_2_SO_4_. Data represent the average value of three experiments. Error bars are the standard deviation (SD) from the mean value

The best pretreatment temperatures for MAG_50_ were 140 °C which resulted in the highest total sugar yield and the lowest FF production (Figs. 2, 4). Therefore, we investigated whether MAG_50_ at 140 °C with a linear decrease in H_2_SO_4_ concentration (Fig. 4) may reduce FF production. Additionally, we assessed the same decreasing effects at 150 °C. When analyzing the data of MAG_50_ at 140 °C, ~ 12% of xylose was missed based on mass–mass balance. Therefore, increasing the H_2_SO_4_ concentration by > 1% is likely to increase this degradation further. Conversely, by reducing the concentration of H_2_SO_4_ from 1 to 0.75% and 0.5%, the total sugar recovery reduced from 81.1 to 71% and 68%, respectively (Fig. 4a, c). A significant reduction in FF production was observed only at an H_2_SO_4_ concentration of 0.5% (Fig. 4a). Pulp yield increased from 55.7 to 64% with no further decrease at 0.5% H_2_SO_4_ (Fig. 4b).

When the acid concentration was linearly decreased from 1 to 0.25% at 150 °C, the total sugar yield gradually reduced from 59 to 57%, 52%, and 46% (Fig. 4a, c). The same trend was observed with the production of FF (Fig. 4a), whereas the residual pulp yield increased gradually (Fig. 4b). Consistent with our previous data, the increase in H_2_SO_4_ concentration accelerated defibrillation resulting in a higher saccharification ratio under optimum conditions [26].

### Individual effects of sulfuric acid, alum, and glycerol and their combined effects during MAG_50_

**Fig. 5:**
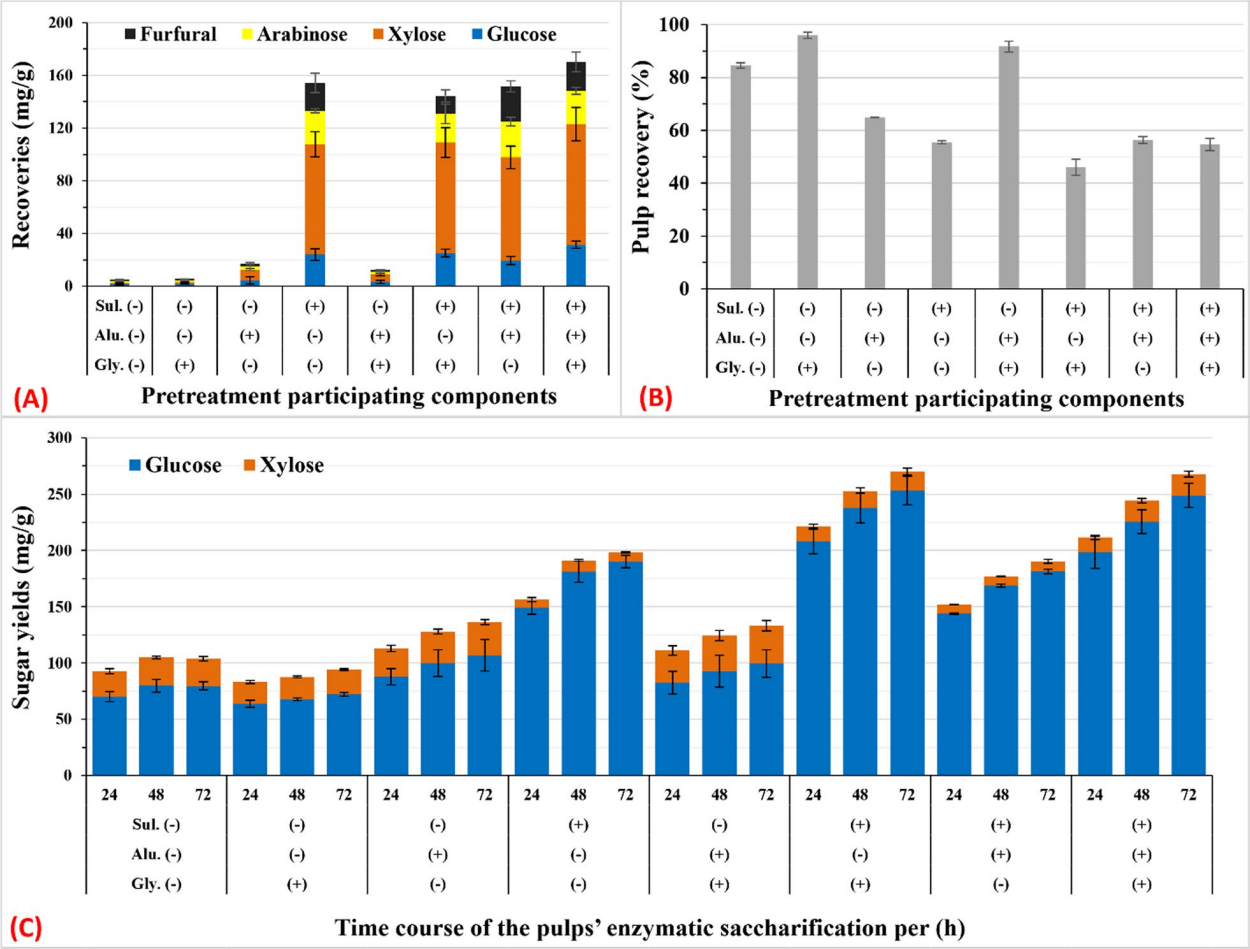
Effects of the combination of glycerol, alum, and sulfuric acid on the concentration of sugar recovery and furfural in GXRS (**A**), pulp yield (**B**), and reducing sugar yield after enzymatic saccharification of pulp with cellulase (10 FPU) (**C**). The pretreatment reaction (15 mL) was performed at 140 °C for 30 min with 1.0 g of SCT with (+) or without (−) 50% aq. glycerol (gly), 360 µM alum (alu), and 1.0% H_2_SO_4_ (sul). Data represent the average value of three experiments. Error bars are the standard deviation (SD) from the mean value

To elucidate the role of each component of the MAG_50_ reaction, the effects of sulfuric acid, alum, glycerol, and microwave heating were examined individually or in combination (Fig. 5). The MAG_50_ reaction, which was fixed at the optimum condition of 140 °C for 30 min as a control, resulted in negligible quantities of sugars after pretreatment (Fig. 5a), although the pulp yield reached 84.5% (Fig. 5b). This was most likely attributable to microwave-accelerated partial delignification (Fig. 5b), although the total sugar recovery after saccharification was 20.5% (Fig. 5a, c). Adding glycerol to the reaction mixture decreased delignification (residual pulp, 94%) and the total sugar yield at 18% (Fig. 5). These data are similar to our previously reported data, wherein 90% glycerol was used for MAG of Japanese cedar and *Paraserianthes falcataria* [27].

Catalysis of alum (Lewis acid, p*K*_a_ ~ 4.9) promoted a decrease in pulp yield to 64.9% (Fig. 5b), subsequently enhancing saccharification and total sugar yield by 40% compared with the control (Fig. 5a, c). Previously, we reported alum as an eco-friendly Lewis acid catalyst at the same dose that can recover the theoretical yield of pulp saccharification at 180 °C of Japanese cedar, *Paraserianthes falcataria*, and *Eucalyptus globulus* [27]; however, the study did not consider the recovery of hemicellulose. H_2_SO_4_ is a strong acid (p*K*_a_ ~ − 4) that contributed significantly to microwave heating during the pretreatment of SCT, resulting in a 63% recovery of the total sugars in SCT (Fig. 5a, c). Consequently, the residual pulp yield decreased to 55.5% (Fig. 5b).

Interestingly, the data regarding the combination of glycerol with alum, H_2_SO_4,_ or with both indicated that glycerol enhanced delignification as well as decreased xylose degradation to FF at a relatively high acid concentration (≤ 1% H_2_SO_4_), while at ≥ 360 µM alum, glycerol inhibited both acid glycerolysis and hydrolysis (Fig. 5). At 1% H_2_SO_4_, 63% of total sugar was recovered and 21 mg/g of FF was produced, whereas at 1% H_2_SO_4_ with 50% glycerol, 76% of total sugar was recovered and 13 mg/g of FF was produced (Fig. 5). Additionally, with the combination of 1% H_2_SO_4_ and alum, 60% of total sugar was recovered and 26 mg/g of FF was produced, whereas with the combination of 1% H_2_SO_4_, alum, and 50% glycerol, 81.1% of total sugar was recovered and 21 mg/g of FF was produced (Fig. 5). These findings provide a basis for further studies on the enhancement of MAG.

### Enhanced saccharification efficiency

**Fig. 6:**
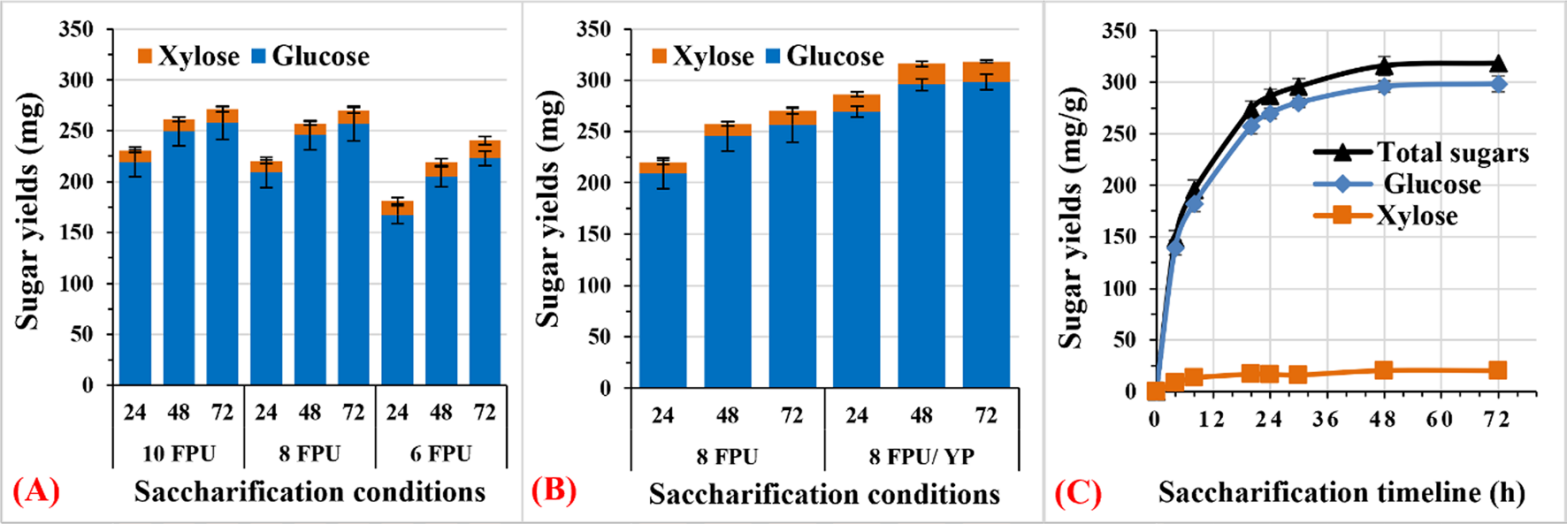
Effects of enzyme dose on reducing sugar yield after enzymatic saccharification of the pulp obtained from ^op^MAG_50_. The saccharification was conducted in a 20 mL solution containing 50 mM succinic acid (pH 5.0), 0.02% sodium azide, and Cellic^®^ CTec2 (6–10 FPU). **A** Effects of the addition of YP on pulp saccharification with cellulase (8 FPU) were evaluated after replacing sodium azide with aureobasidin A (250 µg/L). **B** The time course of saccharification yield using cellulase (8 FPU) and YP (**C**). The pulp was obtained from the best pretreatment reaction at 140 °C for 30 min with 1.0 g of SCT, 50% aq. glycerol, 360 µM alum, and 1.0% H_2_SO_4_. Data represent the average value of three experiments. Error bars are the standard deviation (SD) from the mean value

In this study, we also examined blocking residual lignin binding sites to cellulase enzymes (Fig. 6). Previously, we reported that the addition of yeast extract (10 g/L) and peptone (20 g/L) (YP) accelerated fermentation, particularly during glycerol conversion [28]. Additionally, nonenzymatic proteins such as corn steep liquor, yeast extract, and peptone have enhanced rice straw enzymatic hydrolysis by 12.7%, 13.5%, and 13.7%, respectively [39]. First, residual pulp with different enzyme doses (10, 8, and 6 FPU) did not show significant differences in sugar recovery at 10 and 8 FPU/g of biomass (Fig. 6a). The use of 6 FPU reduced the saccharification yield by 13%, but the saccharification efficiency complemented 83% of the total sugars after 72 h (Fig. 6a). The addition of YP and aureobasidin A as a selective marker for recombinant yeasts to saccharification buffer with 8 FPU of the cellulase enzyme recovered practically the same theoretical glucose. Therefore, YP suppressed the nonproductive binding of cellulase to residual lignin of ^op^MAG_50_ and increased the saccharification efficiency by 18% (Fig. 6b). The addition of YP-enhanced recovery recovers 90% of the saccharification yield after 24 h (Fig. 6c). The accelerating effects of YP can be explained by masking of lignin surfaces, thus protecting the enzyme binding to lignin, as reported for bovine serum albumin [40].

### Bioethanol production

**Fig. 7:**
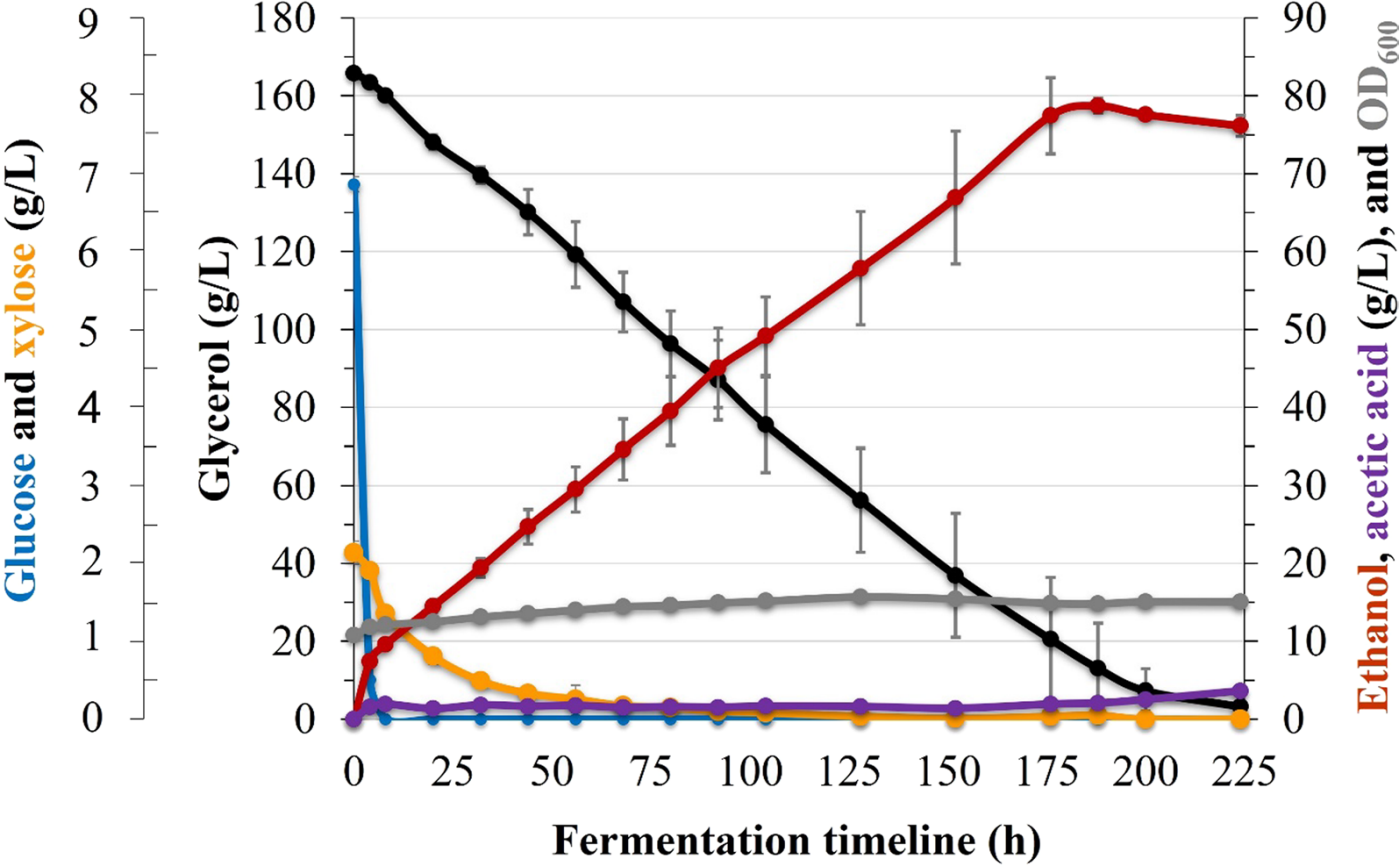
Time-course profiles of SHCF of pulp using glycerol-converting and xylose-fermenting yeasts. Black, blue, orange, red, gray, and purple lines represent glycerol consumption, glucose consumption, xylose consumption, ethanol production, cell density (OD_600_), and acetic acid, respectively. Fermentation was performed in 500-mL Erlenmeyer flasks in triplicate with about 52 mL of liquid culture with orbital shaking at 200 rpm at 30 °C. Data represent the average value of three experiments. Error bars are the standard deviation (SD) from the mean value

Previously, we developed several recombinant yeasts that can ferment xylose [31, 32]. Recently, we also generated *S. cerevisiae* via comprehensive metabolic engineering to efficiently convert glycerol into ethanol [30]. In this study, we examined a mixed culture of xylose and glycerol-fermenting *S. cerevisiae* (Table 2) to produce bioethanol from SCT after ^op^MAG_50_ pretreatment. After separate hydrolysis and co-fermentation** (**SHCF), the engineered strains successfully fermented hydrolyzed glucose and xylose with glycerol. The ethanol productivity reached 78.7 g/L, which corresponded to 10% (v/v) of ethanol with a conversion efficiency of 96% (Fig. 7). Furthermore, we examined simultaneous saccharification and co-fermentation** (**SSCF), and bioethanol production reached 68.6 g/L (8.7% v/v ethanol; Additional file 1: Fig. S1). With co-fermentation of glycerol and xylose, here, the production of ethanol increased in titer yield and efficiency by 6.9 and 1.9-fold, respectively, compared to a previous report, which produced 11.4 g/L ethanol from 55 g/L sugars released by the enzyme saccharification of the acid-pretreated pulp [15]. The expansion of alkali-based ammonia fibers of SCT produced 36 g/L of ethanol after the enzymatic hydrolysis of the 6% pulp using yeast supplementing recombinant xylose [10]. A sequential pretreatment strategy using crude glycerol, ferric chloride, and sodium hydroxide was used to recover the SCT pulp, and the enzyme hydrolysates were fermented to produce 30.4 g/L of bioethanol [7]. Rich cellulose loading after pretreatment with extensive washing has been used to overcome the high titer yield of bioethanol [33, 34]. Here, the co-fermentation of glycerol and sugars after glycerolysis using our recombinant yeasts is a unique alternative to increase the ethanol titer productivity avoiding solvent separation and recovery or increasing the solid loading during saccharification.

**Table 2:** Characteristics of the *S. cerevisiae* strains used in this study

*S. cerevisiae*	Relevant genotypes	References
SK-FGG4	D452-2*, URA3*::* TDH3-OpGDH-DIT1*_*d22*_;* ∆GPD1*::* TDH3-LlNoxE-DIT1*_*d22*_;* AUR1-C*::* PGK*_*p*_*-CuFPS1-RPL41B*_*t*_;* PGK*_*p*_*-ScTPI1-PGK*_*t*_;* PGK*_*p*_*-ScDAK2-PGK*_*t*_;* PGK*_*p*_*-ScDAK1-PGK*_*t*_;* ∆GUT1*::* TEF*_*p*_*-CuFPS1-CYC1*_*t*_;* TYS1*_*p*_*-OpGDH-ATP15*_*t*_;* TDH3-ScDAK1-DIT1*_*d22*_;* FBA1*_*p*_*-ScTPI1-TDH3*_*t*_	[30]
SK-N2	D452-2, *AUR1*::*PGKp-GRE3-PGKt, PGKp-ARSdR-PGKt, PGKp-XK-PGK*	[31]

### Production of AGL agents

In this century, the world has faced several viral epidemics and pandemics. Infectious diseases create fear and health problems that paralyze social activity. Additionally, pathogens attack livestock, leading to significant economic losses. Infections among animals increase the risk of pathogenic viral infections in humans through animal-to-human infection. Therefore, a countermeasure against viral outbreaks is crucial. The virus caused several diseases in animals. The virus was also reported to cause fever in humans after infection [41, 42]. In our previous studies, antiviral substances against EMCV were produced from lignocellulosic biomass by MAG conditions that altered the lignin structure [43–45]. From a biorefinery perspective, the use of whole biomass with fewer processes is important to improve the economic outcome and sustainability. Lignin is the earth’s most abundant natural aromatic polymer, comprising 29% of SCT (Table 1). Therefore, the use of lignin to produce valuable products, in addition to the conversion of sugars to bioethanol, promotes biorefinery applications. Consequently, we surveyed the antiviral activity of ^op^MAG_50_-treated SCT fractions and observed that the acetone extract of the residual pulp exhibited strong antiviral activity against EMCV. The antiviral activity against EMCV was determined by quantifying virus RNA in infected cells using quantitative real-time polymerase chain reaction (PCR). The structure of the acetone extract was analyzed by pyrolysis coupled with gas chromatography interfaced with mass spectrometry ([py-GCMS]; Additional file 1: Fig. S2). The relative molar abundances of the pyrolyzed products are listed in Additional file 1: Table S1. The degradation products were formed from lignin with estimated ratios of H-, G-, and S-units at 39.2%, 41.0%, and 19.8%, respectively. The AGL yield was 7.9% and 27% based on the dry weight of SCT and lignin, respectively.

**Fig. 8:**
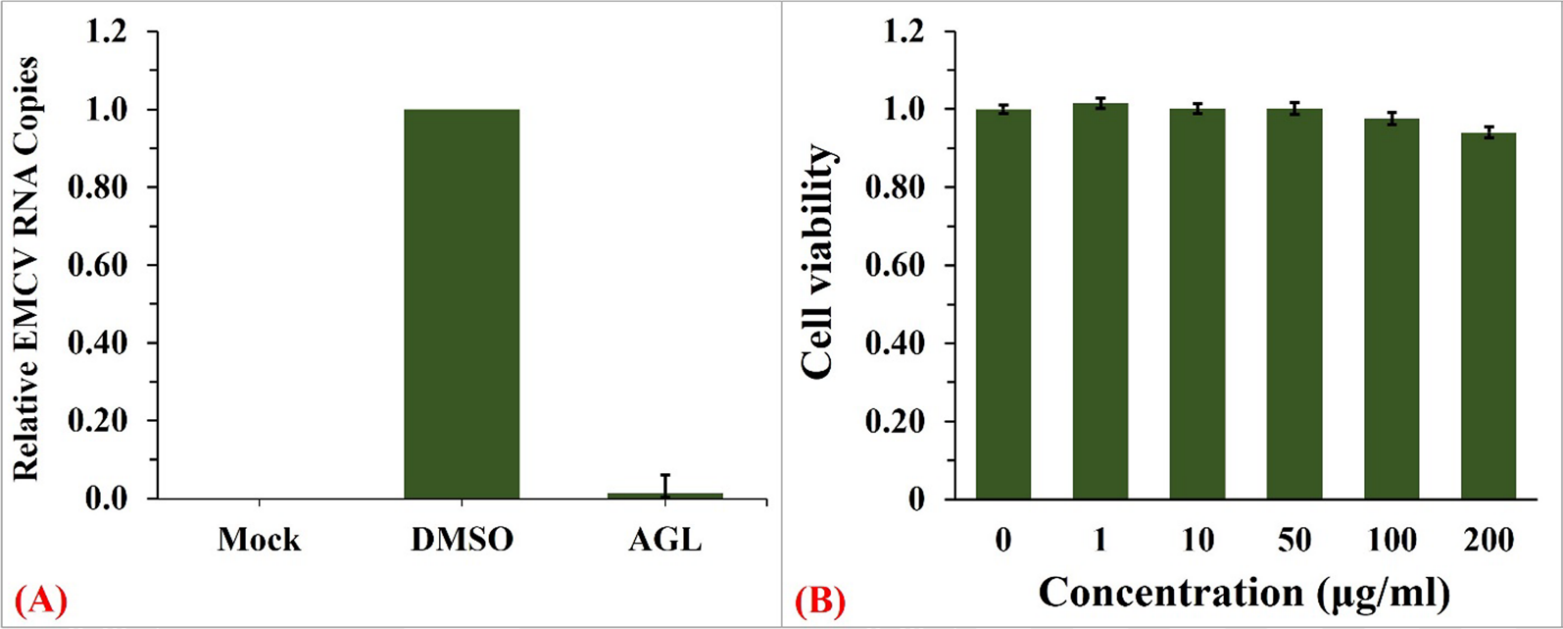
Antiviral activity (**A**) and cytotoxicity (**B**) of AGL. L929 cells were infected with EMCV that had been mixed with 50 mg/mL AGL in DMSO or DMSO without AGL (negative control) and incubated. Relative viral RNA was determined 6 h after infection (**A**). To determine cytotoxicity, L929 cells were incubated for 24 h in DMEM supplemented with designated concentrations of AGL. Cell viability was calculated by counting the cells stained with amido black after incubation with 0, 1, 10, 50, 100, and 200 μg/mL AGL (**B**). Data represent the average of four independent experiments, and error bars represent the standard deviation (SD) from the mean value

AGL inhibited 98.7% of viral replication when the virus was incubated with AGL prior to infection (Fig. 8a). To evaluate cytotoxicity, the viability of L929 cells cultured with AGL was determined by an amido black assay. The antiviral fraction did not inhibit L929 cell growth (Fig. 8b), indicating that the extract has a potent antiviral agent without detectable cytotoxicity. Thus, we established a simple integrated biorefinery system that produces bioethanol and antiviral lignin from SCT via the single-step pretreatment glycerolysis and co-fermentation of glycerol, glucose, and xylose using a mixed culture of two recombinant *S. cerevisiae* strains. Since residual lignin is a potential source of valuable biochemicals that affect saccharification enzyme loading (Fig. 6) and fermentation speed (Fig. 7), further studies to utilize the rest of the acetone-insoluble lignin will promote practical applications of the conversion process.

## Conclusions

In this study, we established a simple integrated biorefinery system for SCT, which facilitates the production of antiviral agents and bioethanol from lignocellulose and glycerol. AGL was extracted after a single-step MAG with acetone from sugarcane pulp in a 7.9% yield of dry SCT (29% of lignin). The residual pulp was enzymatically hydrolyzed to produce reducing sugars with an 89% yield of the total sugars in SCT, and the sugars were co-fermented by mixed cultures of two glycerol- and xylose-fermenting recombinant yeast strains, namely, SK-FGG4, and SK-N2. Using SHCF, an ethanol titer productivity of 78.7 g/L was achieved, corresponding to 10% (v/v ethanol), with a 96% conversion efficiency. To the best of our knowledge, this is the first report on the co-fermentation of glycerol, glucose, and xylose and the integrated production of bioethanol and antiviral agents from SCT. Therefore, the concomitant production in this study may pave the way for the efficient biorefinery of agricultural residues.

## Methods

### Raw materials and chemicals

SCT was obtained from Eastern Sugar and Industries Ltd. (Sa Kaeo, Thailand). It was ground to a length and thickness in the range of approximately 1–8 mm and 0.5–2 mm, respectively. Subsequently, the SCT was used without fractionation or exclusion of any size and stored in double-sealed plastic bags in a dry place at 25 °C ± 2 °C. All chemicals used in this study were of high analytical grade.

### Component analysis by high-performance liquid chromatography (HPLC)

Component analysis was performed using the Prominence HPLC system (Shimadzu, Japan) equipped with refractive index (RID-10A, Shimadzu) and photodiode array (SPD-M20A, Shimadzu) detectors. Aminex HPX-87H (Bio-Rad Laboratories, Hercules, CA, USA) was used to analyze glycerolysis and fermentation products (glucose, glycerol, xylose, ethanol, acetic acid, FF, and HMF) using 5 mM H_2_SO_4_ as an eluent. The RID was used to monitor glucose, glycerol, xylose, ethanol, and acetic acid concentrations. A prominence diode array detector was used to measure FF and HMF concentrations. The peak of xylose overlapped with the peaks of other components in the glycerolysis products obtained from SCT; therefore, Aminex HPX-87P was used to separate the neutral sugars in the pretreated products by elution with H_2_O. Separate components were determined using the RID.

### Compositional analysis of SCT

The water content of the SCT was calculated after drying in an oven at 105 °C. Extracts were removed using a Soxhlet extractor with a mixture of toluene and ethanol (2:1, v/v). The extracts were evaporated and weighed. The extractives-free SCT was recovered and air-dried in a fume hood chamber. The extractives and moisture content were then calculated. Subsequently, the chemical compositions of the SCT and ash content were determined using NREL laboratory analytical procedures [46] with slight modifications. The extractives-free samples (300 mg) were reacted with 72% sulfuric acid (3 mL) at 30 °C for 1 h, followed by dilution to a final concentration of 4% by adding 84 mL of distilled water. The mixture was autoclaved at 121 °C for 1 h, cooled, and filtered through a glass fiber filter (Advantec GA-100, 55 mm, Toyo Roshi Kaisha Ltd., Tokyo, Japan) and a vacuum pump. The resulting soluble fraction was neutralized using potassium hydroxide (pH 6–7) and centrifuged. The supernatant was loaded onto an Oasis HLB cartridge (Waters, Ireland) to remove the hydrophilic lignin that noise analysis using an Aminex column (HPX-87P). Considering that SCT contains > 10% ash, we used the standard method of the Technical Association of the Pulp and Paper Industry to confirm its content. Furthermore, the lignin and holocellulose contents in SCT were determined using the oxidation bleaching method with sodium chlorite–acetic acid, as previously reported [47].

### Pretreatment optimization

SCT (1 g), 50% aq. glycerol (15 mL), H_2_SO_4_ (1%), and alum (360 µm) were mixed by constant stirring in a 100-mL microwave Teflon vessel. After capping, glycerolysis was performed in the range of 100–150 °C for 30 min in a Milestone microwave reactor (StartSYNTH; Microwave Synthesis Labstation, USA). The reaction solution was preheated for 3 min before reaching the maximum temperature, and the cooling time was set at 10 min. The effects of temperature, reaction time, and sulfuric acid concentrations were optimized (Figs. 2, 3, 4, 5). After the reaction and cooling, the mixture was vacuum-filtered with a glass filter to separate the pulp fraction and filtrate. Next, the pulp was washed twice with 10 mL of Milli-Q water, transferred into a 50-mL mighty vial, and extracted twice with 10 mL of acetone to recover the acetone-soluble lignin fraction (i.e., AGL). The GXRS filtrate was weighed and further examined. The residual pulp was washed twice with 10 mL of Milli-Q water to remove the remaining acetone before saccharification.

### Enzymatic saccharification

The wet vacuum-filtered pulp was placed in 100-mL Erlenmeyer flasks with 20 mL sodium succinic buffer (50 mM, pH 5). Sodium azide (20 mg/L) and Cellic^®^ CTec2 (6–10 FPU), indexed at each point, were added to the suspension mixture and incubated at 50 °C by orbital shaking at 150 rpm for 72 h (TAITEC, BioShaker BR-42FL, Japan).

Saccharification experiments were performed using a yeast extract (10 g/L)/peptone (20 g/L) (YP) medium after pretreatment because YP was the preferred fermentation medium in a previous study [30]. Additionally, we replaced sodium azide with 250 µg/L aureobasidin A, an antibiotic that was resistant to the recombinant strains used in this study. During fermentation experiments in the YP medium, the enzyme dosage was decreased to 8 and 6 FPU (Fig. 6). The experiments were performed at least in triplicate. The time course of the hydrolyzed sugar and FF concentrations was determined by HPLC using Aminex columns (HPX-87P or HPX-87H) (Figs. 2, 3, 4, 5, 6).

### Preparation of GXRS for fermentation

^op^MAG_50_ was used to obtain the soluble fraction (GXRS) and pulp from SCT. MAG was performed at 140 °C for 30 min in a solution containing 15 mL of 50% glycerol acidified using 1% sulfuric acid and 360 µM alum/g SCT. After filtration, GXRS was neutralized with KOH (Fig. 1), centrifuged at 15,000 rpm for 5 min, and supplemented with aureobasidin A (250 µg/L), yeast extract (10 g/L), and peptone (20 g/L). The GXRS was filter-sterilized prior to fermentation.

### Cultivation of recombinant yeasts

Here, the engineered *S. cerevisiae*, which efficiently converts glycerol to ethanol (SK-FGG4), was precultured in a solid yeast extract/peptone/dextrose (YPD) medium (Table 2) [30]. Similarly, *S. cerevisiae* (SK-N2) was precultured (Table 2) [31] and cultivated overnight in 10 mL YPD_20_X_20_ medium (20 g/L glucose and 20 g/L xylose). Cell pellets (approximately OD_600_ = 7.7) were collected and used to initiate xylose fermentation in SCT enzymatic hydrolysates. SK-FGG4 was cultured in a YPD_15_G_70_ medium (15 g/L glucose and 70 g/L glycerol), as reported previously [28]. Cell pellets in 150 mL culture medium (approximately OD_600_ = 5) were collected by centrifugation at 5000 rpm. The cell pellets were washed twice using sterile Milli-Q water prior to fermentation.

### SHCF of glycerol and xylose

For bioethanol production, saccharification was performed using Cellic^®^ CTec2/g SCT (8 FPU). Enzymatically hydrolyzed sugars (EHS) were separated from the suspension by centrifugation at 15,000 rpm for 5 min. Next, the EHS was mixed with YP and filter-sterilized before mixing with GXRS (GXRS–EHS, approximately 52 mL). Finally, the cell pellets of the SK-N2 and SK-FGG4 recombinant strains were resuspended in GXRS–EHS and transferred to sterilized 500-mL Erlenmeyer flasks to begin bioethanol production. Fermentation was performed at 30 °C with 150-rpm orbital shaking, and the time course of the conversions was recorded (Fig. 8).

### SSCF of glycerol and xylose

For the SSCF of glycerol and xylose, 50 mM of solid succinic acid buffer was added to GXRS to adjust its pH to 5. Subsequently, 8 mL of the buffered GXRS, corresponding to one-quarter of the total GXRS solution, was added to the saccharification buffer broth containing 20 mL of succinic buffer, 250 µg/L aureobasidin A, cell pellets of SK-N2 and SK-FGG4 recombinant strains (Table 2), and YP before being added into the pulp in 500-mL Erlenmeyer flasks. During SSCF, the other three-quarters of GXRS were batch-fed in three equal dosages of 8 mL, and the conversions were monitored (Additional file 1: Fig. S1).

### Component analysis in fermentation experiments

Glycerol and sugar consumption, ethanol production, and acetic acid secretion were monitored as previously reported [28]. Next, 100 µL of sample obtained from the fermentation flasks was diluted with 900 µL of Milli-Q water in Eppendorf tubes under sterile conditions. The mixture was centrifuged at 15,000 rpm for 5 min, filtered through a 0.45-mm hydrophilic filter (polytetrafluoroethylene), and subjected to HPLC. Peak areas were calculated with high-quality authentic standards according to the following equations:

Efficiency of ethanol production was calculated according to the following equations:

1 $$\begin{aligned} {\text{C}}_{6} {\text{H}}_{12} {\text{O}}_{6} & \to 2{\text{C}}_{2} {\text{H}}_{5} {\text{OH}} + 2{\text{CO}}_{2} \\ 1\,{\text{g}} & \to 0.51\,{\text{g}} + 0:49\,{\text{g}} \\ \end{aligned}$$

Ethanol (grams per liter) = (initial glucose concentration − residual glucose concentration) × 0.51

2 $$\begin{aligned} 3{\text{C}}_{5} {\text{H}}_{10} {\text{O}}_{5} & \to 5{\text{C}}_{2} {\text{H}}_{5} {\text{OH}} + 5{\text{CO}}_{2} \\ 1\,{\text{g}} & \to 0:51\,{\text{g}} + 0:49\,{\text{g}} \\ \end{aligned}$$

Ethanol (grams per liter) = (initial xylose concentration − residual xylose concentration) × 0.51

3 $$\begin{aligned} {\text{C}}_{3} {\text{H}}_{8} {\text{O}}_{3} + {\raise0.7ex\hbox{$1$} \!\mathord{\left/ {\vphantom {1 2}}\right.\kern-0pt} \!\lower0.7ex\hbox{$2$}}{\text{O}}_{2} & \to {\text{C}}_{2} {\text{H}}_{5} {\text{OH}} + {\text{CO}}_{2} + {\text{H}}_{2} {\text{O}} \\ 1\,{\text{g}} & \to 0:5\,{\text{g}} + 0:478\,{\text{g}} \\ \end{aligned}$$

Ethanol (grams per liter) = (initial glycerol concentration − residual glycerol concentration) × 0.50

$${\text{Ethanol volume/volume}}\,\left( \% \right) \, = \frac{{{\text{Concentration}}\,\left( {\text{g/l}} \right) \times 1/\left( {\text{Density of ethanol}} \right)}}{10}.$$

### Production of AGL

After the MAG pretreatment of SCT, lignin degradation products were extracted from the pulp by filtration using acetone in 50-mL vials. Glycerol and xylose concentrations in the acetone solution were determined by HPLC using Aminex HPX-87H. AGL content was calculated by subtracting the weight of glycerol and xylose from the total dry weight of the acetone extract.

### Antiviral assay

L929 mouse fibroblast cells were cultured in Dulbecco’s modified Eagle’s medium (DMEM) supplemented with 10% fetal bovine serum and penicillin–streptomycin (100 units/mL and 100 μg/mL, respectively; DMEM^+^) at 37 °C in a 5% CO_2_ atmosphere. Before the experiments, the cells were seeded in a 12-well plate at 2.5 × 10^5^ cells/well and incubated for 24 h. EMCV was propagated and titrated in Vero cells, and the viral solutions were stored at − 80 °C before use. Crude AGL was prepared as 50 mg/mL dimethylsulfoxide (DMSO) solution after neutralization. Antiviral activity against EMCV was evaluated as previously reported [31]. A mixture of 10 μL AGL solution and 10 μL DMEM containing 1 × 10^6^ pfu/mL EMCV was incubated on ice. Extract-free DMSO was used as the solvent control. After 1 h of incubation, the mixture was diluted with 80 μL of DMEM, and 10 μL of the diluted mixture was added to a well containing L929 cells in 1 mL of DMEM. After 2 h, the culture medium was substituted with 1 mL of DMEM^+^, and incubation continued at 37 °C for another 4 h. Thereafter, total RNA was isolated, and cDNA was prepared using ReverTra Ace qPCR RT Master Mix (Toyobo). EMCV RNA levels were measured using the StepOnePlus real-time PCR system (Applied Biosystems) with the Thunderbird SYBR qPCR Mix (Toyobo). The EMCV-specific primers were as follows: forward 5′-TTA-TAG-TGC-CGG-ACC-TGG-CA-3′ and reverse 5′-CCC-AAG-CTC-CCA-GTG-TTG-TC-3′. The number of EMCV RNA copies was normalized to that of internal β-actin as follows: forward 5′-GAC-ATG-GAG-AAG-ATC-TGG-CAC-CAC-A-3′ and reverse 5′-ATC-TCC-TGC-TCG-AAG-TCT-AGA-GCA-A-3′.

### Cytotoxicity assay

L929 cells were cultured for 24 h in DMEM^+^ containing the antiviral fraction at its designated concentrations. The treated cells were washed thrice with phosphate-buffered saline and fixed with MeOH. After 5 min, MeOH was removed, and a 0.1% (w/v) amido black solution was added for 20 min at 25 °C to stain the cells. The supernatant was removed, and the dye was washed out with 0.1 M NaOH. Its adsorption was then determined by calculating the difference in absorbance at 620 and 405 nm. Cell viability was calculated using the relative absorbance intensity based on the DMSO-treated L929 cells.

### py-GCMS

AGL was washed with distilled water and dried in vacuo prior to py-GCMS. Py-GCMS was performed at 550 °C using a py-2020D pyrolyzer (Frontier Laboratory) connected to a GCMS-QP 2010SE system with a DB-5HT column (30 m × 0.25 mm ID; 0.10-μm film thickness; Agilent Technologies). Helium was used as a carrier gas (1.58 mL/min). The temperature of the GC oven was maintained at 50 °C for 5 min, then increased at 4 °C/min to a final temperature of 280 °C and maintained for 5 min. The pyrolysis components were identified as previously reported [48, 49].

## Supplementary Information

**Additional file 1. Fig. S1**: Simultaneous saccharification and co-fermentation (SSCF) of glycerol and xylose. **Fig. S2**: Total ion chromatogram (TIC) of AGL obtained by py-GCMS using a DB-5HT column. **Table S1**: The relative molar abundances of the aromatic compounds.

## Data Availability

All data are available upon request to the corresponding author.

## Abbreviations

EMCV: Encephalomyocarditis virus
py-GCMS: Pyrolysis coupled with gas chromatography interfaced with mass spectrometry
MAG: Microwave acidic glycerolysis
MAG_50_: MAG with 50% glycerol
^op^MAG_50_: Optimized MAG_50_ (reaction of 1.0 g of SCT/15 mL with 50% aq. glycerol, 360 µM alum, and 1.0% H_2_SO_4_ at 140 °C for 30 min)
AGL: Antiviral glycerolysis lignin
GXRS: Glycerol xylose-rich solution
NADH: Nicotinamide adenine dinucleotide + hydrogen
ES: Enzymatic saccharification
EHS: Enzymatically hydrolyzed sugars
FPU: Filter paper unit
FF: Furfural
HMF: Hydroxymethylfurfural
W/V: Weight/volume
V/V: Volume/volume
YP: Yeast extract (10 g/L) and peptone (20 g/L)
SHCF: Separate hydrolysis and co-fermentation
SSCF: Simultaneous saccharification and co-fermentation
p*K*_a_: Acid dissociation constant
