# Supplementary material for: Efficient integrated production of bioethanol and antiviral glycerolysis lignin from sugarcane trash
Source: Biotechnol Biofuels Bioprod. 2023 May 15;16:82. doi: 10.1186/s13068-023-02333-z (PMC10186800; doi:10.1186/s13068-023-02333-z)
Supplement: Supplementary file 1 — Additional file 1. Fig. S1: Simultaneous saccharification and co-fermentation (SSCF) of glycerol and xylose. Fig. S2: Total ion chromatogram (TIC) of AGL obtained by py-GCMS using a DB-5HT column. Table S1: The relative molar abundances of the aromatic compounds. [file 13068_2023_2333_MOESM1_ESM.docx]

Supporting Information

**Efficient integrated production of bioethanol and antiviral glycerolysis lignin from sugarcane trash**

*Sadat M. R. Khattab ^a, b*^, Hiroyuki Okano ^a^,* *Chihiro Kimura ^a^, Takashi Fujita ^c^ and*

*Takashi Watanabe ^a*^*

(This document contains 3 pages, 1 fig. and 1 table)

^a^ Research Institute for Sustainable Humanosphere, Kyoto University, Gokasho, Uji, Kyoto, 611-0011, Japan

^b^ Faculty of Science, Al-Azhar University, Assiut 71524, Egypt

^c^ Institute for Frontier Life and Medical Sciences, Kyoto University, Shogoin, Kawahara-Cho, Sakyo-Ku, Kyoto, 606-8507, Japan

*khattab.sadatmohamedrezk.7c@kyoto-u.ac.jp; [twatanab@rish.kyoto-u.ac.jp](mailto:twatanab@rish.kyoto-u.ac.jp)


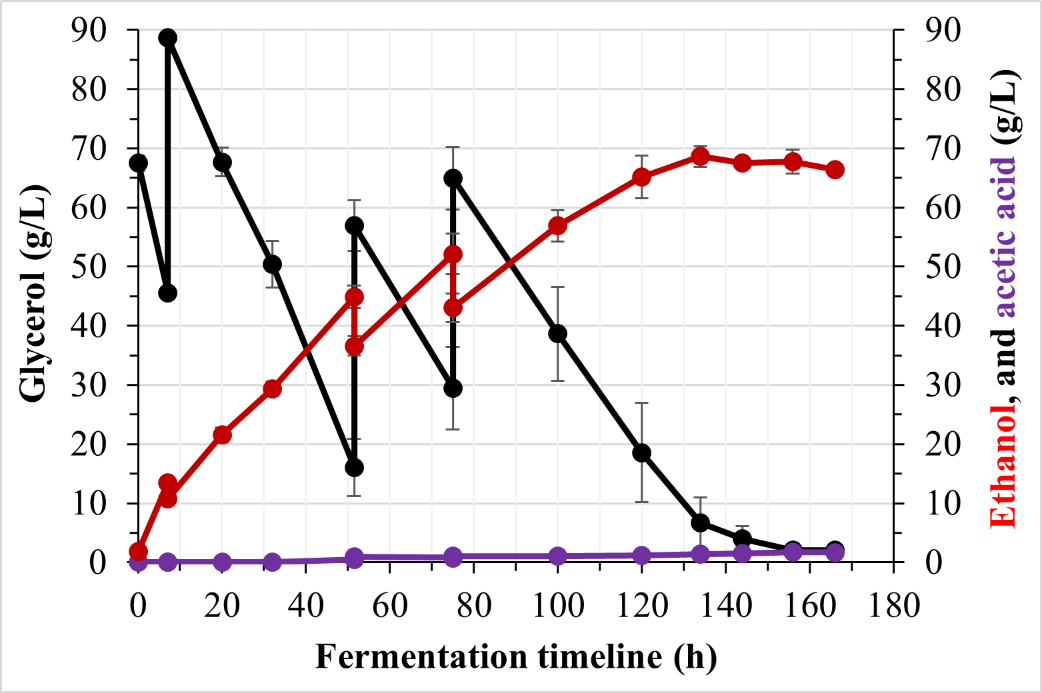


**Fig. S1.** Time-course profiles of SSCF of GXRS using glycerol-converting and xylose-fermenting yeasts. The black, red, and purple lines represent glycerol consumption, ethanol production, and acetic acid. SSCF was conducted in 500-mL Erlenmeyer flasks with orbital shaking at 200 rpm at 30 °C. Data represents the average value of three experiments. Error bars are the standard deviation (SD) from the mean value.

| No. | Compound | Relative molar abundance (%) |
| --- | --- | --- |
| 1 | phenol | 7.9 |
| 2 | methoxytoluene | 0.3 |
| 3 | 2-methylphenol | 1.4 |
| 4 | 4-methylphenol | 2.7 |
| 5 | guaiacol | 11.0 |
| 6 | dimethylphenol | 0.4 |
| 7 | 4-ethylphenol | 4.8 |
| 8 | 4-methylguaiacol | 6.1 |
| 9 | catechol | 2.0 |
| 10 | 4-vinylphenol | 19.7 |
| 11 | 3-methoxycatechol | 2.7 |
| 12 | 4-ethylguaiacol | 4.7 |
| 13 | 4-propylphenol | 0.3 |
| 14 | 4-vinylguaiacol | 12.4 |
| 15 | 2,6-dimethoxyphenol | 5.1 |
| 16 | 4-propylguaiacol | 0.3 |
| 17 | vanillin | 0.5 |
| 18 | *trans*-isoeugenol | 2.4 |
| 19 | 2,6-dimethoxy-4-methylphenol | 2.7 |
| 20 | apocynin | 0.9 |
| 21 | 2,6-dimethoxy-4-ethylphenol | 1.1 |
| 22 | guaiacylacetone | 0.7 |
| 23 | 2,6-dimethoxy-4-vinylphenol | 2.5 |
| 24 | 2,6-dimethoxy-4-allylphenol | 0.6 |
| 25 | 2,6-dimethoxy-4-propylphenol | 0.3 |
| 26 | *cis*-2,6-dimethoxy-4-propenylphenol | 0.4 |
| 27 | *trans-*2,6-dimethoxy-4-propenylphenol | 2.2 |
| 28 | acetosyringone | 1.9 |
| 29 | syringylacetone | 1.0 |
| 30 | propiosyringone | 1.0 |

Table S1. Relative molar abundance of aromatic compounds detected by py-GCMS of AGL

All H-, G-, and S-derived peaks were used for calculation of the lignin-derived core unit ratio, except for methoxytoluene (peak 2), catechol (peak 9), and 3-methoxycatechol (peak 11).
